# The Measurement of Aesthetic Emotion in Music

**DOI:** 10.3389/fpsyg.2017.00651

**Published:** 2017-05-03

**Authors:** Leon Crickmore

**Affiliations:** RetiredLondon, UK

**Keywords:** enjoyment of music, growth in appreciation, evolution of music, factor analysis, aesthetic emotion, dynamic gestalt

In an earlier Opinion Article, Perlovsky challenged cognitive scientists to measure aesthetic emotions (Perlovsky, 2014). The present author claims to have done exactly that almost 50 years ago (Crickmore, 1968, 1973). Details of supportive factor analyses (Thomson, 1951) may be viewed in the attached Supplementary Material.

## A syndrome test of music appreciation

My syndrome hypothesis serves both as a functional definition of music appreciation, and as an operational definition of a dynamic gestalt. The hypothesis states that when a musical composition has been assimilated aesthetically, it will leave the listener feeling interested, happier, more relaxed, with a desire to remain quiet, satisfied and without any particular mental pictures. To cite a typical example of such a response from a student:

Record 5 (Whistlin' Rufus: Chris Barber's Jazz Band)

L7^1^, I+, M+, T−, V−, S+, P0

“Mind completely at rest and relaxed. Very enjoyable piece of music.”

The psychological experience indicated by this syndrome may differ in intensity according to the profundity of the music, or to the listener's emotional state at the time. Occasionally, the experience can even reach the sublimity which T. S. Eliot describes in the third of his *Four Quartets:*

“Music heard so deeply

That it is not heard at all, but you are the music

While the music lasts.”

Lest any readers should consider that such an intense experience would be unlikely to be within the grasp of an 18-year-old technical student, let me quote an instance from a student. After listening to a recording of the second movement of Tchaikovsky's Sixth Symphony—a piece which another student described as the “worst record played yet”—this student responded with the full syndrome, adding:

“I wasn't listening to the music but it was *me* inside. I was living it–this was especially noticeable with the climaxes.”

Various factor analyses of my experimental data were carried out. These showed the Syndrome Test to be independent of the Maudsley Personality Inventory, the Wing Standardized Test of Musical Intelligence and Raven's Progressive Matrices (A–E)—a non-verbal test related to general intelligence. Also, when a number of the musical items were repeated later, the increase in the scores of students proved to be statistically significant, indicating that the test can also be used to measure growth in appreciation. This feature raises an issue that may be interpreted as a demonstration of the statistical unreliability of the test, as has been suggested by Clive Gabriel in our joint article (Gabriel and Crickmore, 1977). This article describes the first use of the test with a totally random population, rather than with a group of students who had chosen to participate in a course of musical appreciation. Alternatively, such a result could be cited as evidence of the inappropriateness of traditional psychometric methodology for measuring the formation of dynamic gestalten, since such a process involves the integration of sub-wholes at a moment of ripeness (Maritain, 1959, pp. 132–134)^2^. The most frequent pattern of responses which Gabriel found was the postulated profile (G) with the exception of an incorrect response on scale P (P+ instead of P−). He called this response “a relaxed G.” Yet on another occasion one of my students after listening to the first movement of Beethoven's Fifth Symphony wrote:

“It is surprising too that when one's mind ‘wanders’ into mental pictures the actual beauty of the music escapes one and only surface music is absorbed.”

## Aesthetic emotions

Perlovsky states that “emotions related to knowledge have been called aesthetic since the time of Kant.” I would contend, however, that since Kant held knowledge to be exclusively “discursive,” that is to say the opposite of receptive and contemplative (Pieper, 1959, p. 32), this dogmatic assertion has contributed to the formulation of a methodology in psychology which has constrained cognitive scientists from attempting to measure aesthetic emotions. This postulate led Kant (1790) to rank music in the “lowest place” amongst the arts, and Pinker (1997) to dismiss music as “mere auditory cheese.” Cross (1999, 2013) and Bannon (2016), however, both cite evidence indicating that Darwin believed, but deemed it inappropriate to say so at the time, that music's origins preceded those of language, which later emerged as an offshoot.

The concept of aesthetic emotion in academic literature seems to have been first posited by the aesthetician Bell (1914) and incorporated into cognitive psychology by Payne (1961, 1965). Later it was developed philosophically by Langer (1957). Langer's argues that music is both “a symbolism without assigned connotation” and “our myth of the inner life,” a view that is wholly compatible with my syndrome hypothesis. Nevertheless, I prefer the greater metaphysical precision of the older explanation offered by Thomas Aquinas who distinguished between conceptual knowledge (*per cognitionem*) and knowledge from experience (*per connaturalitatem*). The aesthetic enjoyment of music belongs to the latter category (Crickmore, 1966).

Probably the best psychological theory that matches such philosophical views lies in Gestalt psychology, in the concept of a “dynamic gestalt,” as expounded by Perls, Hefferline and Goodman's in *Gestalt Therapy* (Perls et al., 1951, pp. 403–404). Their book maps a process of contact, conceived as a single whole but which can also be conveniently divided into a series of grounds and figures: Fore-contact; Contacting; Final contact—when a listener would be “all ears” (Crickmore, 1972)—and Post-contact.

## Musical emotions

Cooke (1959) has described music as a “language of the emotions,” and has demonstrated how certain tonal patterns have become conventionally associated with particular basic emotions. The idea that an aesthetic response to music can be defined as a match between the emotions of a composer and those of the listener is widely accepted. However, people do in fact feel different emotions when listening to the same music, while a single listener may feel different emotions when listening to the same music at different times. I suggest, therefore, that musical emotions which do not lead to physical action are best considered as “as if” emotions. Langer has noted (Langer, 1957, p. 238), that music reflects “only the morphology of feeling.” Musical emotions are more a matter of stipulation rather than of logical necessity.

Despite all that, a number of cognitive psychologists have assembled evidence for the measurability of a number of basic musical emotions (Juslin, 2013) With a view to future research, I have therefore added a means for registering these in a revised form of my Syndrome Test. The Revised Syndrome Test is shown in Table 1. The test in its original form is displayed in the upper part of the Table, with the exclusion of the opportunity for listeners to record their “Liking” for the music on a scale of 1–7. This was omitted, when it was discovered how highly the L. scale correlated with the I. scale. A new lower part of the test now provides opportunities for listeners to record the musical emotions they experience.

**Table 1:** **Revised syndrome test**.

** +**	**0**	**−**		**Responses**
I Interested	Indifferent	Bored		
M Happier	No change	Sadder		
T More tense	No change	More relaxed		
V Desire to talk	No change	Desire to remain quiet		
S Satisfied	No change	Confused		
P Mental pictures	Name them:			
			None 0	
			Some +	
Any other comments:				
Then please tick any of the emotions listed below which you associate with the music you have just heard, and describe any others you have felt but which are not listed:				
	**Tick √**			
	Joy			
	Sadness			
	Love			
	Anxiety			
	Anger			
	Pride			
	Any other:			

*This revision of the syndrome test is characterized by the addition of a possibility for each listener to tick emotions felt during the music from a list of six emotions for which researchers have already found validating evidence. In this form the test has not yet been used experimentally. Until experiment provides some initial evidence concerning which emotions listeners actually do tick, and how frequently, it is not possible to decide whether, and if so how, such fresh data might be incorporated into a Factor Analysis of scores on the scales of the syndrome, together with those from some appropriate personality test. If those who believe that music is in some way a language of the emotions are right, such an analysis should be feasible. If my hypothesis that music is not a language of the emotions is true, any emerging data is unlikely to be compatible with factor analysis*.

It has subsequently been found that the empirically verifiable musical emotions in several different cultures tend to be conventionally associated with a particular musical genre—for example, sadness—funeral music; pride—a national anthem. During the evolution of each of these genres, it seems likely that music would initially have been introduced as part of a communal ritual, thereby heightening the intrinsic emotions of a particular social activity. Only later, would a few well-manicure examples of these genres have come to be listened to in contemplative silence. Recently, some evidence has been found for a link between musical genre and personality (Neuman et al., 2016).

This evolutionary consideration brings to mind two even more significant pieces of recent pioneering research, the first by Perlovsky (2008, 2010, 2014) and Nikolsky (2015, 2016). For Perlovsky music has played a far greater part in human evolution than has so far been generally recognized. He defines its main evolutionary function as the resolution of the cognitive dissonances brought about by the split in the vocalizations of proto-humans into two types, one evolving into language, and the other into music. He also postulates the existence of a “knowledge instinct” through which the brain is able to match top-down and bottom-up signals in a manner for which he has coined the term “dynamic logic.” Furthermore, his dynamic logic is computable, and could well share something in common with the psychological idea of a dynamic gestalt.

Working within Perlovsky's theoretical framework, and employing a methodology for melodic analysis little known outside the world of Russian-speaking musicologists, Nikolsky has managed to define, together with appropriate audio illustrations, 11 stages in the evolutionary development of music, from the time of Stephen Mithen's “Singing Neanderthals” (Mithen, 2005) and the earliest pre-modal music, via pentatonic and heptatonic stages and culminating in modern tonality. Could it be that, just as Pierre Teilhard de Chardin proposed (de Chardin, 1959; Crickmore, 1978), unlike the majority of modern scientists who tend to consider the biosphere as being the result of a largely fortuitous series of events, human evolution has a direction: namely the establishment of a phylum of “direct cerebralization?” Once this threshold of refection has been crossed evolution would continue in a new form, that of humanly invented combinations—logic, mathematics, art, and music. Could it also be possible that Nikolsky's model of tonal evolution might be taken to suggest a direction for musical evolution: toward the establishment of the diatonic system? Gary Tomlinson's *A Million Years of Music* (Tomlinson, 2015) is probably the best available survey of current knowledge in this field.

Each age in the evolution of music would have required a different style of listening. For us, in the twenty-first century, any description of this must take account of the effects of popular music with all its associated digitization and consumerist technologies. Our traditional definition of music in the West, based as it is largely on the world of concert halls and opera-houses therefore needs to be extended, possibly even to include listening to a Walkman and Muzak. Philip Tagg's book *Music's Meanings* (Tagg, 2013) could help us clear away some of the intellectual brushwood which has been accumulated over the years by musicologists and historians of music. Tagg has suggested an operational re-definition of music as “that form of human communication in which humanly organized non-verbal sound can, following culturally specific conventions, carry meaning relating to emotional, gestural, tactile, kinetic, spatial and prosodic patterns of cognition.”

## Conclusion

There is a need for a programme of further research using my Revised Syndrome Test in Table 1. Such a programme should seek to clarify further the nature of the relationships between aesthetic emotions as measured by my test, musical emotions, genres, and personality. It should also include some fMRI scanning of the participants, both as they enjoy familiar music and as they grow to enjoy that which is less familiar to them. As long as Homo sapiens survive, music will survive too. In his poem *The Metalogue to the Magic Flute*, W. H. Auden has highlighted a matter which future researchers will have to bear in mind:

“The history of music as of Man

Will not go cancrizans, and no ear can

Recall what, when Archduke *Francis* reigned,

Was heard by ears whose treasure-hoard contained

A *Flute* already but as yet no *Ring*,

Each age has its own mode of listening.”

## Ethics statement

The experiment mentioned took place in the 1960s before the Helsinki agreement with the consent of the students involved.

## Author contributions

The author confirms being the sole contributor of this work and approved it for publication.

### Conflict of interest statement

The author declares that the research was conducted in the absence of any commercial or financial relationships that could be construed as a potential conflict of interest.

## Footnotes

^1^Liking 7 or 6 was included in the earliest versions of the test but later discarded.

^2^I would justify the inclusion in the factor analyses of derived scores, and rotation to discover the most helpful viewing point on the grounds that loadings are simply mathematical entities (entia rationis).
