# Supplementary material for: The Measurement of Aesthetic Emotion in Music
Source: Front Psychol. 2017 May 3;8:651. doi: 10.3389/fpsyg.2017.00651 (PMC5413555; doi:10.3389/fpsyg.2017.00651)
Supplement: Supplementary file 1 [file DataSheet1.DOCX]

**The measurement of aesthetic emotion in music**

**Supplementary material**

**Table A**

**General factor with the highest loadings on scale G.** Scale G refers to the total number of complete syndromes achieved by each student. These totals included the G scores from the repetition of certain musical items. To some extent, therefore, G scores may be taken to be reflecting in part the students’ hypothetical growth in music appreciation. Thomson (Thomson, 1951) contains a detailed mathematical explanation of Factor Analysis. A Centroid Analysis is the basic form for dealing with a multiple of factors. With regard to the interpretation of the factor loadings, theoretically, 1·0 would indicate identity between two sets of scores and 0 their complete independence. By convention the decimal point is sometimes omitted in Tables of loadings. Factors can usually be identified from their highest loading. The usual reason for rotating factors is to maximize the number of positive loadings. But in this case, it was done to find the best viewpoint from which to highlight the investigator’s intuitive syndrome hypothesis.

|  |  |  |  |  |  |  |
| --- | --- | --- | --- | --- | --- | --- |
|  |  | *1960-1* | *1961-2* | *1968-9* | *1969-70* |  |
|  | L | 34 | 06 | - | - |  |
|  | I | 43 | 06 | 61 | 62 |  |
|  | M | 45 | 42 | 49 | 60 |  |
|  | T | 54 | 79 | 65 | 61 |  |
|  | V | 65 | 74 | 65 | 22 |  |
|  | S | 62 | 21 | 65 | 76 |  |
|  | P | 23 | 59 | 21 | 11 |  |
|  | G | 92 | 82 | 77 | 85 |  |
|  |  |  |  |  |  |  |

**The measurement of aesthetic emotion in music**

**Supplementary material**

**Table B**

**The bi-polarity of scale P:** A bi-polar factor is one which has a positive loading on one particular scale combined with a negative loading on another. This implies that the presence of the positive item is normally accompanied by the absence of the negative one. Thus in the case of scale G (the syndrome), it will be noticed that an otherwise complete syndrome can often be accompanied by an incorrect response on scale P (mental pictures) – a phenomenon for which Gabriel (Gabriel and Crickmore, 1977) coined the term ‘relaxed syndrome’. Varimax and Promax refer to alternative kinds of rotation. The former helps to differentiate between the variables; the latter to identify oblique factors related to clusters of variables. Such forms of analysis turned out to be particularly appropriate for dealing with the syndrome data, since, in the light of the gestalt axiom that the unfragmented whole is greater than the sum of its parts, the inclusion of the dependent G scores had been permitted^[[1]](#footnote-1)^.

|  |  |  |  |  |  |
| --- | --- | --- | --- | --- | --- |
|  |  | *Analysis* | *Factor* | *Per cent  of variance* |  |
|  | G | 1960-1 | Varimax II | 31·1 |  |
|  |  | 1961-2 | Varimax II | 30·2 |  |
|  |  | 1968-9 | Promax I | 36·1 |  |
|  |  | 1969-70 | Varimax I | 35·4 |  |
| **P/rest of syndrome**   \|  \| \| --- \| |  | 1960-1 | Varimax I | 36·5 |  |
|  |  | 1961-2 | Varimax I | 34·3 |  |
|  |  | 1968-9 | Varimax I | 38·1 |  |
|  |  | 1969-70 | Promax I | 55·2 |  |
|  |  |  |  |  |  |

**The measurement of aesthetic emotion in music**

**Supplementary material**

**Table C**

|  |  |  |  |  |  |  |  |  |  |  |
| --- | --- | --- | --- | --- | --- | --- | --- | --- | --- | --- |

**Rotated Loadings for all tests 1960-2:** Key: * = Highest loadings. L = Liking. I = Interest. M = Happiness. T = Relaxation. V = Desire to remain quiet. S = Satisfaction. P = Absence of pictures. G = Number of syndromes. E = Extroversion. N = Neuroticism. W = Musical ability. R = Intellectual capacity. X = Increase (Growth). Panel **A** shows the test-loadings for 1960-1 and Panel **B** those for 1961-2. The numbering of the 1960-1 components (I–V), which are to be interpreted as Factors, has been reordered in the 1961-2 data to indicate the best possible match between the two years. The five columns can be interpreted as factors, identified by means of their highest positive loadings. For example, in both Panels the loading of 0.94 for test R (Ravens Progressive Matrices (A-E) – a non-verbal test of intellectual capacity) – is clearly the Factor represented by component V in both Panels. In Panel **B** the re-ordering of the numbering I-V from Panel **A** simply shows the different order in which the components emerged during this analysis. But as the identification of the factors from the highest positive loadings makes clear, the five columns in the order presented indicate the most likely match between the factors involved. Thus, for example, the third column, component III in Panel **A** and component II in Panel **B**, can be identified from its highest positive loadings (0.80) as representing test G (the number of syndromes). Negative loadings make the interpretation of the factors more difficult. My guess is that the relatively high number of negative loadings might have been caused by so many different kinds of tests being analysed together. In general, however, the negative loadings in Table 5 can be explained either as an indicator of a bi-polarity between the syndrome and some other single test or cluster of tests, or as an indicator of the bi-polarity between test E (Extroversion) and N (Neuroticism). The latter bi-polarity constitutes a particularly puzzling phenomenon, since theoretically Eysenck’s two factors should be totally independent. Perhaps the adolescence of the student population has contributed to this anomaly. The relatively high loadings in each of the Panels between test X (Increase/Growth), E (Extroversion) and N (Neuroticism) are of considerable interest. The relationship of growth both to musical choice and to personality is an area for further research.

|  |  |  | **A 1960-1** | |  |  |  |  |  | **B 1961-2** | |  |  |  |
| --- | --- | --- | --- | --- | --- | --- | --- | --- | --- | --- | --- | --- | --- | --- |
|  | Expanded Rotated Loadings 1960-61 | | | | | |  | Expanded Rotated Loadings 1961-62 | | | | | |  |
|  | *Test* | *I* | *II* | *III* | *IV* | *V* |  | *Test* | *I* | *IV* | *II* | *III* | *V* |  |
|  | L | 86* | 8 | 23 | 13 | -16 |  | L | 78* | 2 | 17 | 30 | -3 |  |
|  | I | 84* | 7 | 38 | 3 | 9 |  | I | 91* | -5 | 9 | 1 | 13 |  |
|  | M | 70* | -23 | 22 | 35 | 4 |  | M | 28 | -26 | 63* | 38 | -7 |  |
|  | T | 30 | -5 | 25 | 73* | 12 |  | T | 12 | -1 | 78* | -17 | -8 |  |
|  | V | 27 | 29 | 68* | 3 | -11 |  | V | 19 | 27 | 81* | 6 | 3 |  |
|  | S | 60* | -15 | 35 | 46 | 34 |  | S | 74* | -17 | 30 | 19 | -9 |  |
|  | P | -76* | -3 | 38 | -10 | -9 |  | P | -5 | 9 | 28 | -84* | -2 |  |
|  | G | 16 | -29 | 80* | 33 | 9 |  | G | 12 | 0 | 80* | -16 | -16 |  |
|  | E | -4 | -73* | -27 | 10 | -10 |  | E | -60* | -39 | -7 | 45 | 1 |  |
|  | N | 14 | 75* | -31 | 26 | 4 |  | N | -8 | 88* | 7 | 2 | -11 |  |
|  | W | 7 | 1 | -1 | 88* | -4 |  | W | 24 | 33 | 15 | 67* | 13 |  |
|  | R | 13 | 0 | -2 | 2 | 94* |  | R | 7 | -13 | -13 | 7 | 94* |  |
|  | X | 35 | -65* | 4 | 19 | 30 |  | X | -48 | 42 | -22 | 16 | 46 |  |
|  | **Percent** | **24,75** | **13,72** | **14,32** | **14,5** | **9,01** |  | **Percent** | **21,65** | **10,8** | **20,12** | **13,13** | **9,06** |  |
|  |  |  |  |  |  |  |  |  |  |  |  |  |  |  |
|  |  |  |  |  |  |  |  |  |  |  |  |  |  |  |

v

1. I would justify this inclusion as follows: in the 1960s, we believed that factors corresponded to innate human abilities. Now the preferred view is that loadings are no more than mathematical entities (Maritain, (Maritain, 1959: 132-134) would have described these as *entia rationis),* which signify relationships between loadings as seen from some particular position. I believe, therefore, that I am justified in using the rotation of factors to a desirable position and the inclusion of derived scores in order to locate the best possible viewing point for interpreting the likely significance of the factors. [↑](#footnote-ref-1)
